# Three New Sesquiterpene Aryl Esters from the Mycelium of *Armillaria mellea*

**DOI:** 10.3390/molecules20069994

**Published:** 2015-05-29

**Authors:** Chien-Chih Chen, Yueh-Hsiung Kuo, Jing-Jy Cheng, Ping-Jyun Sung, Ching-Li Ni, Chin-Chu Chen, Chien-Chang Shen

**Affiliations:** 1 Department of Biotechnology, HungKuang University, Sha Lu, Taichung 433, Taiwan; E-Mails: ccchen@sunrise.hk.edu.tw (C.-C.C.); alyssum15@yahoo.com.tw (C.-L.N.); 2 Department of Nursing, HungKuang University, Sha Lu, Taichung 433, Taiwan; 3 Department of Chinese Pharmaceutical Sciences and Chinese Medicine Resources, China Medical University, Taichung 404, Taiwan; E-Mail: kuoyh@mail.cmu.edu.tw; 4 Department of Biotechnology, Asia University, Taichung 413, Taiwan; 5 National Research Institute of Chinese Medicine, Ministry of Health and Welfare, Peitou, Taipei 112, Taiwan; E-Mail: verona@nricm.edu.tw; 6 Graduate Institute of Marine Biology, National Dong Hwa University, Pingtung 944, Taiwan; E-Mail: pjsung@nmmba.gov.tw; 7 National Museum of Marine Biology and Aquarium, Pingtung 944, Taiwan; 8 Biotechnology Center, Grape King Bio Ltd., Chung Li, Taoyuan 320, Taiwan; E-Mail: gkbioeng@grapeking.com.tw

**Keywords:** sesquiterpene aryl esters, *Armillaria mellea*, Tricholomataceae, cytotoxicity

## Abstract

Three new sesquiterpene aryl esters and eight known compounds were isolated from the EtOH extract of the mycelium of *Armillaria mellea*. The structures of new compounds were established by analysis of their spectroscopic data. Some of the isolates showed cytotoxicity to a variety of cancer cell lines, including MCF-7, H460, HT-29, and CEM.

## 1. Introduction

*Armillaria mellea* (Tricholomataceae) is a fungus symbiotic with the Chinese medicinal herb “Tianma” (*Gastrodia elata* Blume). The fruiting bodies of *A. mellea* have been used in Traditional Chinese Medicine for the treatment of hypertension, headache, insomnia, dizziness, and vertigo. Recently, the cultured mycelium of *A*. *mellea* became a health food in Taiwan and China and its tablets are used to treat geriatric patients with palsy, headache, insomnia, dizziness, and neurasthenia [1,2,3,4,5]. Previous chemical studies of *A*. *mellea* reported the isolation of a number of sesquiterpene aromatic esters, in which the tricyclic 5-6-4 protoilludane or protoilludene alcohols were esterified with orsellinic acid or its derivatives [1,2,3,6,7,8,9,10,11,12]. Some of the sesquiterpene aryl esters exhibited cytotoxic activities against human cancer cells. Among these sesquiterpene aryl esters, arnamial showed cytotoxicity against MCF-7, CCRF-CEM, HCT-116, and Jurkat T cells, but melledonal C only showed cytotoxic activity against CCRF-CEM cells [11]. Armillaridin was reported to exhibit cytotoxicity against MCF-7, HeLa, K562, and Jurkat T cells [12]. In addition to inhibiting the growth of the cancer cells, armillaridin also enhanced radiosensitivity of human esophageal cancer cells and there might be potential to integrate armillaridin with radiotherapy for esophageal cancer treatment [13]. Moreover, 4-*O*-methylarmillaridin showed cytotoxicity against MCF-7 and Jurkat T cells and dehydroarmillyl orsellinate showed cytotoxicity against MCF-7 and K562 cells [12]. Recently, armillarikin was reported to inhibit growth and induce apoptosis in human leukaemic K562, U937, and HL-60 cells [14]. In this paper, we describe the isolation and structural elucidation of three new sesquiterpene aryl esters from the mycelium of *A*. *mellea*, as well as the cytotoxic activities of the isolated components [15].

## 2. Results and Discussion

**Figure 1:**
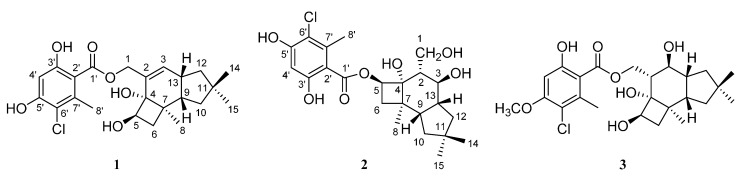
The structures of **1**–**3** isolated from the mycelium of *Amillaria mellea*.

The EtOH extract of the mycelium of *A. mellea* was partitioned with EtOAc and H_2_O. The EtOAc layer was chromatographed repeatedly to afford three new compounds, **1**–**3** (Figure 1), along with eight known compounds: 6′-chloromelleolide F (**4**) [16], 13-hydroxymelleolide K (**5**) [17], armillaricin (**6**) [3], armillaridin (**7**) [1], armillarikin (**8**) [2], melleolide F (**9**) [11], melledonal C (**10**) [18], and melledonal B (**11**) [18]. The structures of these new compounds were established by their spectroscopic data and the known compounds were identified by comparison of their NMR data with those reported in the literature.

**Figure 2:**
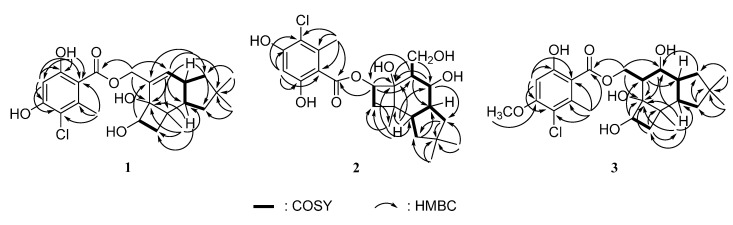
COSY and selected HMBC correlations of **1**–**3**.

Compound **1** was obtained as a colorless solid. Its ESIMS spectrum showed two peaks at *m*/*z* 459 and 461, which were attributed to [M + Na]^+^ and [M + 2 + Na]^+^ ions, respectively. The ratio of their intensities was about 3:1, suggesting the presence of a chlorine atom in compound **1**. The molecular formula C_23_H_29_ClO_6_ was deduced from FABHRMS and NMR (Table 1) spectra, which indicated that there were nine double bond equivalents. The ^13^C and DEPT spectra displayed four methyl carbons at δ 19.7, 22.3, 32.1, and 32.3, four methylene carbons, including an oxymethylene group at δ 67.8, five methine carbons, including one oxymethine group at δ 76.9 and two olefinic or aromatic carbons at δ 102.7 and 136.1, and ten quaternary carbons. Four of the quaternary carbons were oxygenated where one carbonyl, one aliphatic, and two aromatic carbons showed signals at δ 170.4, 78.1, 158.2, and 161.8, respectively. The ^1^H-NMR spectrum of **1** exhibited four methyl singlets at δ 0.95, 0.97, 1.15, and 2.62 and oxymethylene signals at δ 4.93 (dt, *J* = 12.6, 1.2 Hz) and 5.19 (ddd, *J* = 12.6, 1.8, 0.6 Hz). Two singlets at δ 5.87 and 6.43 were derived from olefinic or aromatic protons. Moreover, a signal at δ 10.74 revealed the presence of a chelated phenolic hydroxyl group. The one bond ^1^H–^13^C connectivities were analyzed by using HSQC data and protons attached to carbons were assigned. In the COSY spectrum of **1**, proton H-9 at δ 2.12 showed correlations with H_2_-10 (δ 1.35 and 1.47) and H-13 (δ 2.73) and proton H-13 also showed correlation with H_2_-12 (δ 1.83), which indicated a linkage of C-10–C-9–C-13–C-12 (Figure 2). Besides, the linkages of C-3–C-13 and C-5–C-6 were deduced by COSY cross-peaks of H-3/H-13 and H-5/H_2_-6. Further connectivities were established by long-range HMBC correlations shown in Figure 2. Cross-peaks of H-3/C-4,C-9,C-12 and H-13/C-2,C-7,C-10,C-11 revealed that a cyclopentane ring was fused to a cyclohexene ring. Moreover, HMBC cross-peaks of H-5/C-2,C-4,C-6 and H-9/C-4,C-6 as well as the COSY correlation of H-5 and H_2_-6 suggested that a cyclobutane ring was fused to the cyclohexene ring. The HMBC data further showed cross-peaks of H_3_-8/C-4,C-6,C-7,C-9 and H_3_-14,H_3_-15/C-10–C-12, which indicated that one and two methyl groups were attached to C-7 and C-11, respectively. Besides, the correlations of H_2_-1 to C-2, C-3 and C-4 suggested that an oxygenated methylene group was linked to C-2. Based on the above evidence, a protoillud-7-ene skeleton was deduced for compound **1** [6]. In addition to one double bond in the protoilludene moiety, six other unsaturated carbon signals and a total of nine double bond equivalents in **1** revealed that an aromatic ring could be a part of this compound. Furthermore, HMBC cross-peaks of H-8′/C-2′,C-6′,C-7′ and H-4′/C-2′,C-3′,C-5′,C-6′ were observed, which suggested the presence of a 3-chloro-4,6-dihydroxy-2-methylphenyl moiety. In the HMBC spectrum acquired in CDCl_3_, the proton signal (δ 11.08) of the hydroxyl group chelated to the carbonyl group at the aromatic ring showed correlations with C-2′, C-3′, and C-4′, which indicated that the hydroxyl and the carbonyl groups were located at C-3′ and C-2′, respectively. Hence, the chlorine atom was suggested to be located at C-6′. The linkage of this benzoyl group to the oxygen atom at C-1 was confirmed by the HMBC correlation of H_2_-1 to C-1′. The relative configuration of **1** was deduced from NOE experiments in CDCl_3_. The irradiation of H_3_-8 enhanced the signals of H-5 and H-6a (δ 1.87); however, the irradiation of H-9 enhanced the signals of H-6b (δ 1.30), H-13, and H_3_-14. Thus, H-6b, H-9, H-13, and H_3_-14 were on the same face of the protoilludene moiety and H-5, H-6a, and H_3_-8 were on the other face. In the ^13^C-NMR spectrum acquired in CDCl_3_, signals for C-2–C-15 resembled those of a protoilludene-type sesquiterpene, echinocidin B, isolated from a mycelial culture of *Echinodontium tsugicola*, in which *cis* junctures of cyclohexene to both cyclobutane and cyclopentane rings were determined [19]. Accordingly, it was deduced that the cyclohexene ring in **1** was also fused to both cyclobutane and cyclopentane rings in a *cis*-fashion and the relative structure of **1** was established. Compound **1** was given the trivial name melleolide N.

**Table 1:** ^1^H- and ^13^C-NMR data of compounds **1**–**3** in acetone-*d*_6_^a^.

Position	1		2		3	
	δ_H_^b^	δ_C_	δ_H_^b^	δ_C_	δ_H_^b^	δ_C_
1	4.93 (dt, 12.6, 1.2) 5.19 (ddd, 12.6, 1.8, 0.6)	67.8	3.89 (dd, 10.8, 4.8) 4.03 (dd, 11.4, 3.6)	62.9	4.68 (dd, 11.4, 4.2) 4.71 (dd, 11.4, 4.2)	66.2
2		132.0	2.09 (m)	46.4	2.40 (dt, 10.8, 4.2)	43.8
3	5.87 (br s)	136.1	3.72 (t, 11.4)	69.0	3.77 (t, 10.8)	68.6
4		78.1		81.4		81.8
5	4.33 (t, 8.4)	76.9	5.33 (t, 8.4)	77.2	4.19 (t, 8.4)	73.4
6	1.38 (dd, 10.8, 9.0) 1.75 (dd, 10.8, 8.4)	36.4	1.85 (dd, 10.8, 9.0) 1.95 (dd, 10.8, 7.8)	34.5	1.54 (dd, 10.2, 8.4) 1.71 (dd, 10.8, 8.4)	37.0
7		38.0		39.0		37.7
8	1.15 (s)	22.3	1.16 (s)	22.3	1.06 (s)	22.5
9	2.12 (m)	45.4	2.16 (m)	48.1	2.09 (m)	48.3
10	1.35 (dd, 12.6, 7.2) 1.46 (t, 12.6)	42.3	1.46 (m)	44.4	1.44 (m)	44.7
11		38.5		36.7		36.7
12	1.45 (d, 13.2) 1.83 (dd, 13.2, 8.4)	48.3	1.52 (dd, 13.8, 7.8) 1.98 (d, 14.4)	43.4	1.52 (dd, 13.8, 7.8) 1.97 (m)	43.6
13	2.73 (br t, 7.8)	40.2	2.00 (m)	47.5	1.97 (m)	47.9
14	0.97 (s)	32.3	0.99 (s)	32.4	0.99 (s)	32.4
15	0.95 (s)	32.1	1.09 (s)	32.7	1.07 (s)	32.7
1′		170.4		171.1		169.0
2′		109.0		108.3		110.4
3′		161.8		162.4		160.9
4′	6.43 (s)	102.7	6.45 (s)	102.6	6.45 (s)	99.6
5′		158.2		158.6		159.4
6′		114.7		114.8		115.2
7′		140.4		140.2		139.8
8′	2.62 (s)	19.7	2.62 (s)	19.9	2.57 (s)	18.9
3′-OH	10.74 (br s)		10.97 (br s)			
5′-OH	9.42 (br s)		9.56 (br s)			
5′-OCH_3_					3.89 (s)	55.6

^a^ Spectra recorded at 600 MHz for ^1^H-NMR and 150 MHz for ^13^C-NMR; ^b^ Multiplicities and *J* values (in Hz) are in parentheses.

Compound **2** gave a molecular formula of C_23_H_31_ClO_7_, which was deduced from its ESIHRMS and NMR (Table 1) spectra. Its ^13^C and DEPT NMR spectra showed four methyl at δ 19.9, 22.3, 32.4, and 32.7, four methylene, six methine, and nine quaternary carbons. In addition to a carbonyl group with a signal at δ 171.1, six carbons were oxygenated, which included one oxymethylene signal at δ 62.9, two oxymethine signals at δ 69.0 and 77.2, one aliphatic quaternary carbon signal at δ 81.4, and two aromatic carbon signals at δ 158.6 and 162.4. The carbon signals above 100 ppm were almost the same as the signals of 3-chloro-4,6-dihydroxy-2-methylbenzoyl group in **1**, suggesting the presence of this benzoyl group in **2**. The ^1^H-NMR spectrum of **2** exhibited four methyl groups at δ 0.99, 1.09 (6H), and 2.62. Besides, it showed two signals at δ 3.89 (dd, *J* = 10.8, 4.8 Hz) and 4.03 (dd, *J* = 11.4, 3.6 Hz), which correlated to the carbon signal at δ 62.9 in the HSQC spectrum and were attributed to an oxymethylene group. Moreover, two signals at δ 3.72 (t, *J* = 11.4 Hz) and 5.33 (t, *J* = 8.4 Hz) correlating to the carbon signals at δ 69.0 and 77.2, respectively, indicated the presence of two oxymethine groups. A signal at δ 6.45 (s) was also observed and the proton correlated to the carbon resonating at δ 102.6, suggesting that they were in an aromatic ring. Its HMBC spectrum (Figure 2) displayed cross-peaks of H_2_-1/C-2–C-4, H-3/C-1,C-4,C-12, H-5/C-2,C-4,C-6, H_3_-8/C-4,C-6,C-7,C-9, and H_2_-12/C-3,C-10,C-11,C-13–C-15, which revealed a protoilludane skeleton for **2** with C-1, C-3, C-4, and C-5 oxygenated. The ^13^C-NMR data of the protoilludane moiety in CD_3_OD were almost the same as those in 5′-methoxy-6′-chloroarmillane [12], which suggested that the structures of the protoilludane moieties in **2** and 5′-methoxy-6′-chloroarmillane were the same. The linkage of 3-chloro-4,6-dihydroxy-2-methylbenzoyl group to the oxygen atom at C-5 was confirmed by the HMBC correlation of H-5 to C-1′. In the NOESY spectrum, the cross-peaks of H_3_-14/H-9, H-12b (δ 1.52), H-9/H-6b (δ 1.85), and H-6b/H-2 revealed that H-2, H-6b, H-9, H-12b, and H_3_-14 were on the same face of the protoilludane moiety. Cross-peaks of H-3/H_3_-15,H-12a (δ 1.98), H_3_-8/H-5,H-6a (δ 1.95) indicated that H-3, H-5, H-6a, H_3_-8, H-12a, and H_3_-15 were on the other face. Furthermore, the large coupling constant of 11.2 Hz for H-3 indicated that H-2 and H-3 were in a *trans* configuration and the hydroxy-methyl and 3-OH groups were on opposite faces. Thus, the relative structure of **2** was determined and it was named melleolide Q. This compound is similar to 5′-methoxy-6′-chloroarmillane where a methoxyl group instead of a hydroxyl group is located at C-5′ [12].

Compound **3** gave a molecular formula of C_24_H_33_ClO_7_ deduced from FABHRMS and NMR (Table 1) spectra. Its ^13^C-NMR spectrum displayed 24 signals, including a methoxyl signal at δ 55.6 and a carbonyl signal at δ 169.0, and the signals for C-3, C-4, and C-8–C-15 were similar to those in **2**. In the aromatic region, the signals resembled those in **2** except that the signal for C-4′ shifted from δ 102.6 to 99.6. In the HMBC spectrum (Figure 2), the methoxyl protons showed correlation to C-5′, which indicated that the methoxyl group was attached to C-5′ carbon of the aromatic ring. The ^1^H-NMR spectrum of **3** was also similar to that of **2** except for the signals of H_2_-1, H-2, H-5, and H_2_-6 as well as one additional signal at δ 3.89 (s) attributed to a methoxyl group. Signals for H_2_-1 shifted downfield to δ 4.68 (dd, *J* = 11.4, 4.2 Hz) and 4.71 (dd, *J* = 11.4, 4.2 Hz) from δ 3.89 and 4.03; signals for H-2 shifted downfield to δ 2.40 (dt, *J* = 10.8, 4.2 Hz) from δ 2.09. Moreover, H-5 signal shifted upfield from δ 5.33 to 4.19 (t, *J* = 8.4 Hz) and the signals of H_2_-6 shifted upfield from δ 1.85 and 1.95 to δ 1.54 (dd, *J* = 10.2, 8.4 Hz) and 1.71 (dd, *J* = 10.8, 8.4 Hz). Therefore, the benzoyloxy group in **3** was suggested to be linked to C-1, which was confirmed by the HMBC correlation of H_2_-1 to C-1′. The stereochemistry of **3** was established by the NOESY spectrum and the coupling constant of H-2 and H-3. The NOESY cross-peaks of H_3_-14/H-9,H-12b (δ 1.52), H-9/H-6b (δ 1.54), H-3/H-12a (δ 1.97), and H-5/H_3_-8,H-6a (δ 1.71) and the large coupling constant of 10.8 Hz for H-3 revealed that its relative configuration was the same as that in **2**. Accordingly, the relative structure of **3** was determined and it was named melleolide R.

The isolated compounds from *A*. *mellea* were tested *in vitro* for cytotoxicity to a variety of human cancer cell lines including MCF-7, H460, HT-29, and CEM and the results are summarized in Table 2. Compounds **2**–**4** and **6**–**9** showed cytotoxicity to MCF-7 cells, in which compound **2** was most cytotoxic. Compounds **1**, **4**, and **6**–**9** exhibited comparable cytotoxicity against H460 cells. Compounds **1** and **6** showed stronger cytotoxicity to HT-29 cells than other tested compounds. Compounds **1**, **3**, and **5**–**7** showed comparable cytotoxicity to human leukemia cells. Among all tested compounds, **6** exhibited cytotoxicity to all of these cancer cells. Compounds **1**–**5** and **7**–**9** showed selective cytotoxicity and compounds **10** and **11** were inactive to these cancer cell lines.

**Table 2:** Cytotoxicity of the isolated compounds from *A*. *mellea* against several cancer cell lines ^a^.

Compound	IC_50_ (μM)			
	MCF-7	H460	HT-29	CEM
**1**	56.5 ± 4.2	5.5 ± 0.6	7.1 ± 0.8	5.4 ± 0.3
**2**	1.5 ± 0.1	80.0 ± 8.9	54.2 ± 4.7	10.3 ± 2.3
**3**	3.7 ± 0.3	53.8 ± 6.2	18.7 ± 3.2	3.4 ± 0.2
**4**	4.8 ± 0.5	4.5 ± 0.4	56.7 ± 4.5	28.8 ± 1.2
**5**	>100	>100	32.1 ± 3.6	5.5 ± 0.6
**6**	4.8 ± 0.4	5.5 ± 0.4	4.6 ± 0.3	5.8 ± 0.6
**7**	1.7 ± 0.2	4.5 ± 0.3	42.1 ± 5.1	5.1 ± 0.4
**8**	4.4 ± 0.8	5.7 ± 0.5	34.7 ± 4.6	44.6 ± 4.4
**9**	8.3 ± 2.2	5.1 ± 0.2	58.4 ± 4.9	41.2 ± 3.4
**10**	>100	>100	85.6 ± 9.1	49.6 ± 5.2
**11**	>100	>100	>100	>100
**Dox**	0.27 ± 0.02	0.01 ± 0.005	0.12 ± 0.01	0.09 ± 0.01

^a^ MCF-7, human breast cancer; H460, human lung cancer; HT-29, human colon cancer; CEM, human leukaemia. Dox: Doxorubicin as a positive control.

## 3. Experimental Section

### 3.1. General Experimental Procedures

Optical rotations were taken on a P-2000 digital polarimeter (JASCO, Tokyo, Japan). UV spectra were measured on a U-3310 spectrophotometer (Hitachi, Tokyo, Japan). IR spectra were recorded on an Avatar 320 FT-IR spectrophotometer (Nicolet, Madison, WI, USA). ^1^H-, ^13^C-, and 2D-NMR spectra were recorded on a VNMRS 600 MHz spectrometer (Varian, Palo Alto, CA, USA). ESIMS and HRMS spectra were obtained on LCQ and Quest MAT 95XL spectrometers (Finnigan/Thermo, San Jose, CA, USA), respectively. HPLC was conducted on a model 1100 system (HP, Palo Alto, CA, USA) equipped with a G1311A QuatPump, a G1322A degasser, and a G1315B photodiode array detector set at 254 nm. Semipreparative HPLC was performed using a reversed-phase column (Cosmosil 5C_18_-MS-II, 5 μm, 10 × 250 mm) at a flow rate of 2.0 mL/min.

### 3.2. Source of Organism

The strain of the fungus *A.* *mellea* (# BCRC 36361) was purchased from the Food Industry Research and Development Institute, Hsinchu, Taiwan.

### 3.3. Fermentation of Organism

The strain BCRC 36361 was inoculated into 1 L of the medium (1.0% glucose, 1.0% oat powder, 0.1% peptone, 0.1% yeast extract, pH 4.5) in a 2-L Hinton flask at 25 °C on a rotary shaker (120 rpm) for six days. The mycelium was aseptically transferred to a 500-L fermenter containing 400 L of the above medium and incubated at 25 °C for ten days.

### 3.4. Extraction and Isolation

The mycelium of *A*. *mellea* (9.0 kg) was extracted with 95% EtOH (50 L) three times. The 95% EtOH soluble portion was concentrated to give the EtOH extract, which was partitioned with H_2_O and EtOAc. The EtOAc layer was chromatographed on silica gel column and eluted with *n*-hexane-EtOAc (20:1 → 0:1) to provide ten fractions (Fr-1–Fr-10). Fraction Fr-3 (*n*-hexane/EtOAc = 5:1) was concentrated and recrystallized in MeOH to afford armillaricin (**6**, 114 mg). Fraction Fr-4 (*n*-hexane/EtOAc = 5:1) was purified by semi-preparative reversed-phase HPLC [H_2_O/CH_3_CN (15:85, 0 min) → H_2_O/CH_3_CN (0:100, 20 min)] to give armillaridin (**7**, 365 mg, Rt = 18.53 min). Fraction Fr-7 (*n*-hexane/EtOAc = 1:1) was repeatedly chromatographed on silica gel (CHCl_3_/MeOH = 50:1 → 20:1) and Sephadex LH-20 (MeOH) columns to yield armillarikin (**8**, 676 mg), **2** (42.9 mg), 6′-chloromelleolide F (**4**, 57.7 mg), and melleolide F (**9**, 37 mg). Fraction Fr-9 (*n*-hexane/EtOAc = 0:1) was repeatedly chromatographed on silica gel (CHCl_3_/MeOH = 30:1 → 20:1), Sephadex LH-20 (H_2_O/MeOH = 3:7), and RP-18 (H_2_O/MeOH = 2:8) columns to afford two compounds, melledonal C (**10**, 3.50 g) and **1** (101 mg), and one sub-fraction Fr-9-1. Sub-fraction Fr-9-1 was further purified by chromatography on a RP-18 column (H_2_O/MeOH = 2:8) to give **3** (18 mg). Fraction Fr-10 (*n*-hexane/EtOAc = 0:1) was repeatedly chromatographed on silica gel (CHCl_3_/MeOH = 30:1 → 15:1), Sephadex LH-20 (MeOH), and RP-18 (H_2_O/MeOH = 3:7) columns to afford 13-hydroxymelleolide K (**5**, 595 mg) and melledonal B (**11**, 118 mg).

#### 3.4.1. Melleolide N (**1**)

Colorless powder; ${\left[\text{α}\right]}_{D}^{25}$ −25 (*c* 0.05, MeOH); UV (MeOH) λ_max_ (log ε) 307 (3.65), 261 (3.91), 213 (4.45) nm; IR (KBr) ν_max_ 3528, 1631, 1592, 1461, 1425, 1310, 1243, 1128, 1081 cm^−1^; ^1^H- and ^13^C-NMR data: see Table 1; ESIMS *m*/*z* (%) 459 [M + Na]^+^ (100), 461 (36); FABHRMS *m*/*z* 437.1732 [M + H]^+^ (calcd for C_23_H_30_ClO_6_ [M + H]^+^, 437.1731).

#### 3.4.2. Melleolide Q (**2**)

Colorless powder; ${\left[\text{α}\right]}_{D}^{25}$ −70 (*c* 0.05, MeOH); UV (MeOH) λ_max_ (log ε) 310 (3.44), 263 (3.78), 215 (4.20) nm; IR (KBr) ν_max_ 3400, 2950, 2868, 1655, 1608, 1313, 1241, 1160, 1099, 1029 cm^−^^1^; ^1^H- and ^13^C-NMR data: see Table 1; ESIMS *m*/*z* (%) 477 [M + Na]^+^ (100), 479 (33); ESIHRMS *m*/*z* 453.1684 [M – H]^−^ (calcd for C_23_H_30_ClO_7_ [M − H]^−^, 453.1675).

#### 3.4.3. Melleolide R (**3**)

Colorless powder; ${\left[\text{α}\right]}_{D}^{25}$ –10 (*c* 0.06, MeOH); UV (MeOH) λ_max_ (log ε) 303 (3.61), 258 (3.95), 216 (4.47) nm; IR (KBr) ν_max_ 3500, 2951, 2863, 1648, 1600, 1453, 1366, 1319, 1240, 1101, 1042 cm^−1^; ^1^H- and ^13^C-NMR data: see Table 1; FABMS *m*/*z* (%) 469 [M + H]^+^; FABHRMS *m*/*z* 469.1991 [M + H]^+^ (calcd for C_24_H_34_ClO_7_ [M + H]^+^, 469.1993).

### 3.5. Cell Culture

Four cancer cell lines, MCF-7, H460, HT-29, and CEM, were derived from the American Type Culture Collection (Manassas, VA, USA) and were maintained in DMEM or RPMI medium supplemented with 2 mM L-glutamine and 10% heat-inactivated fetal bovine serum (FBS) under standard culture conditions. The cell viability and cell number were determined by the Trypan Blue dye-exclusion method.

### 3.6. Cancer Cell Cytotoxicity Assay

To assess cell viability, the alamar blue (AB) assay (dye purchased from Biosource International, Nivelles, Belgium) was used as previously described [20]. This involved aspirating medium at the end of each treatment period and adding 100 µL of fresh medium containing 10% *v*/*v* AB to control and treated wells. Plates were incubated at 37 °C for 6 h prior to measuring the absorbance at 540 nm and at 595 nm wavelengths using a spectrophotometric plate reader. Experimental data were normalized to control values.

## 4. Conclusions

Three new sesquiterpene aryl esters, melleolides N (**1**), Q (**2**), and R (**3**), together with eight known compounds **4**–**11** were isolated from the EtOH extract of the mycelium of *Armillaria mellea*. Nine isolates showed cytotoxicity to a variety of cancer cell lines, including MCF-7, H460, HT-29, and CEM. Among the isolates, compound **6** exhibited cytotoxicity to all of these cancer cells and compounds **1**–**5** and **7**–**9** showed selective cytotoxicity.
